# Analytical investigation of Carreau fluid flow through a non-circular conduit with wavy wall

**DOI:** 10.1038/s41598-024-52848-0

**Published:** 2024-01-29

**Authors:** Muhammad Hasnain Shahzad, Aziz Ullah Awan, Ali Akgül, Sohail Nadeem, Kamel Guedri, Murad Khan Hassani, Basim M. Makhdoum

**Affiliations:** 1 Department of Mathematics, University of the Punjab, Lahore, 54590 Pakistan; 2 Department of Computer Science and Mathematics, Lebanese American University, Beirut, Lebanon; 3 Department of Mathematics, Art and Science Faculty, Siirt University, 56100 Siirt, Turkey; 4 Department of Mathematics, Mathematics Research Center, Near East University, Near East Boulevard, 99138 Nicosia, Mersin 10, Turkey; 5 Department of Mathematics, Quaid-i-Azam University 45320, Islamabad, 44000 Pakistan; 6 Department of Mathematics, Wenzhou University, Wenzhou, China; 7 Mechanical Engineering Department, College of Engineering and Islamic Architecture, Umm Al-Qura University, P.O. Box 5555, 21955 Makkah, Saudi Arabia; 8 Department of Mathematics, Ghazni University, Ghazni, Afghanistan

**Keywords:** Engineering, Mathematics and computing

## Abstract

Peristaltic flow through an elliptic channel has vital significance in different scientific and engineering applications. The peristaltic flow of Carreau fluid through a duct with an elliptical cross-section is investigated in this work . The proposed problem is defined mathematically in Cartesian coordinates by incorporating no-slip boundary conditions. The mathematical equations are solved in their dimensionless form under the approximation of long wavelength. The solution of the momentum equation is obtained by applying perturbation technique ($W_e^2$ as perturbation parameter) along with a polynomial solution. We introduce a new polynomial of twenty degrees to solve the energy equation. The solutions of mathematical equations are investigated deeply through graphical analysis. It is noted that non-Newtonian effects are dominant along the minor axis. It is found that flow velocity is higher in the channels having a high elliptical cross-section. It is observed from the streamlines that the flow is smooth in the mid-region, but they transform into contours towards the peristaltic moving wall of the elliptic duct.

## Introduction

The sinusoidal progression of the conduit walls causes peristaltic motion. Peristaltic transport is caused by a progressive sinusoidal wave that moves with the channel’s boundary. The movement of food through the large intestine is an example of peristalsis. The area of its applications is vast and broad. It has applications in the industrial, physiological, and engineering fields^1^. The industrial processes in which the peristaltic mechanism is utilized involve several biomedical devices, such as dialysis machines, blood-pumping machines, heart-lung machines, ortho pumps, and corrosive and sanitary liquid flows. The heart-lung machine specifically enables the cardiovascular bypass, in which, during surgery, the machine overcomes the functionality of the heart and lungs. Food movement, urine transport, the esophagus, and blood circulation in small blood vessels are all examples of applications in physiology.

The exciting and vital applications of the peristaltic mechanism attracted the researcher towards itself. Many researchers have investigated the peristaltic flow through circular ducts. Mekheimer worked on the peristaltic flow of fluid through a circular tube under the magnetic field effects by considering the couple stresses^2^. Nadeem and Akram examined the Williamson fluid’s peristaltic transport and solved the non-linear model of differential equations using an analytical technique. They studied the effect of various physical constraints in the model on the flow^3^. Tiripathi and Baig investigated the nanofluid flow analytically through a 2-D channel with wavy walls, which has applications in drug delivery to the digestive system^4^. Ashraf et al. studied the peristaltic cilia-produced movement of developing the embryo from the ampulla to the human fallopian tube via intramural. They used the perturbation technique to handle the Johnson-Segalman fluid model^5^. Tiripathi used the fractional model of Oldroyd-B fluid to analyze the peristaltic movement of chyme in the small intestine, and he worked on the homotopy analysis method to get the solution of differential equations^6^.

Ellahi et al. discussed the bioheat and mass transfer in peristaltic flow through a rectangular duct of a non-uniform cross-section. They briefly analyzed the physical parameter’s impact on the flow and studied the trapping phenomenon^7^. Zeeshan et al. examined the dusty fluid flow with Casson fluid (biorheological fluid) as the base fluid under the magnetic impact. They discussed the effects of parameters, especially the quantity of nanoparticles and the magnetic parameter on the flow^8^. Nadeem et al. used the eigenfunctions to solve the mathematical model representing the water-based nanofluid flow through a rectangular channel and analyzed the flow properties like fluid’s velocity and its thermal conductivity^9^. More literature on the peristaltic flow is provided in^10–12^. Recently, some researchers have gained more interest in the fluid flow via ducts of elliptical cross-sections. Saleem et al. studied the peristaltic transport of Casson fluid in an elliptical duct. They examined the impact of critical physical parameters on the nature of velocity, temperature, and pressure-rise profiles^13^. Rachid et al. investigated the mechanical efficiency and entropy production of peristaltic transport of Casson fluid via the elliptic duct^14^.

As mentioned earlier, the literature shows that the peristaltic flow of Carreau fluid in an elliptic duct has yet to be investigated. Therefore, in the current work, we considered the flow of Carreau fluid through a duct of the elliptical cross-section with a sinusoidally moving wall. The partial differential equations representing the problem are solved by using the perturbation technique. We also utilized the polynomials of degrees four and twenty to get the solution to mathematical equations. Then these solutions are examined graphically in detail for various physical constraints of the study.

## Mathematical formulation

**Figure 1:**
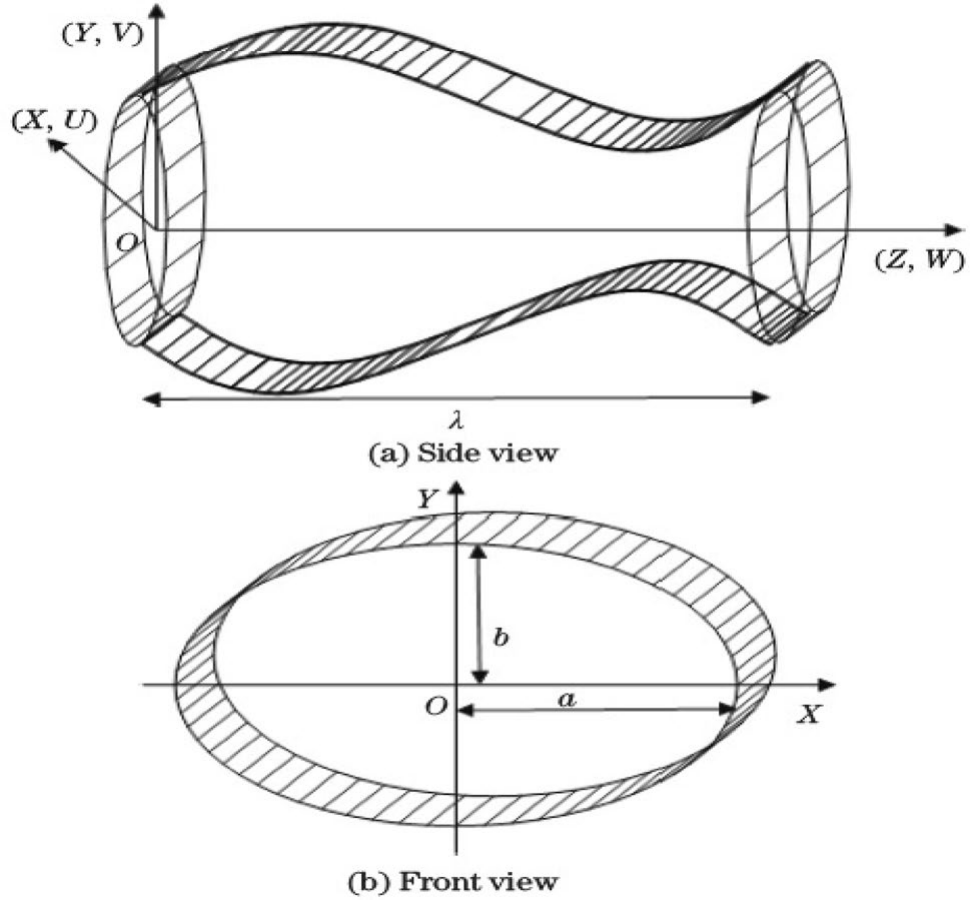
Geometry of the problem.

Consider the flow of incompressible non-Newtonian fluid through the duct of an elliptic cross-section having a sinusoidally moving boundary wall. The Carreau fluid model is accounted for the non-Newtonian characteristics of the fluid. Further, the fluid is considered to have no slip at the peristaltically moving wall of the elliptic conduit. The proposed problem is studied in Cartesian coordinates, and its geometrical representation is given in Fig. 1.

The sinusoidally fluctuating boundary wall of elliptic conduit with *a* as the semi-major axis and *b* as the semi-minor axis can be represented mathematically as

1 $$\begin{aligned} b\left( Z,t\right)&=b_0+ d\sin \left( \frac{2\pi }{\lambda }\left( Z-ct\right) \right) , \end{aligned}$$

2 $$\begin{aligned} a\left( Z,t\right)&=3\, b\left( Z,t\right) , \end{aligned}$$

where $b_0$ is the semi-minor axis of a non-deformed elliptic channel.

The mathematical equations that govern the non-Newtonian incompressible fluid are^15^

3 $$\begin{aligned} \partial _{X}U+\partial _{Y}V+\partial _{Z}W&=0, \end{aligned}$$

4 $$\begin{aligned} \rho \left( \partial _{t}U+U\,\partial _{X}U+V\,\partial _{Y}U+W\,\partial _{Z}U\right)&=-\partial _{X}P +\partial _{X}\Lambda _{XX}+\partial _{Y}\Lambda _{XY}+\partial _{Z}\Lambda _{XZ},\end{aligned}$$

5 $$\begin{aligned} \rho \left( \partial _{t}V+U\,\partial _{X}V+V\,\partial _{Y}V+W\,\partial _{Z}V\right)&=-\partial _{Y}P +\partial _{X}\Lambda _{YX}+\partial _{Y}\Lambda _{YY}+\partial _{Z}\Lambda _{YZ},\end{aligned}$$

6 $$\begin{aligned} \rho \left( \partial _{t}W+U\,\partial _{X}W+V\,\partial _{Y}W+W\,\partial _{Z}W\right)&=-\partial _{Z}P+\partial _{X}\Lambda _{ZX}+\partial _{Y}\Lambda _{ZY}+\partial _{Z}\Lambda _{ZZ},\end{aligned}$$

7 $$\begin{aligned} \rho c_p\left( \partial _{t}T+U\,\partial _{X}T+V\,\partial _{Y}T+W\,\partial _{Z}T\right)&=k\left( \partial _{XX}T+\partial _{YY}T+\partial _{ZZ}T\right) \nonumber \\&\quad +\,\left( \Lambda _{XX}\partial _{X}U+\Lambda _{XY}\partial _{Y}U+\Lambda _{XZ}\partial _{Z}U\right) \nonumber \\&\quad +\,\left( \Lambda _{YX}\partial _{X}V+\Lambda _{YY}\partial _{Y}V+\Lambda _{YZ}\partial _{Z}V\right) \nonumber \\&\quad +\,\left( \Lambda _{ZX}\partial _{X}W+\Lambda _{ZY}\partial _{Y}W+\Lambda _{ZZ}\partial _{Z}W\right) . \end{aligned}$$

The corresponding boundary conditions over the elliptic cross-section are

8 $$\begin{aligned} W=0,\,\,\, \,\,\, T=T_w\,\,\, \,\,\, \text {at}\,\,\, \frac{X^{2}}{a^{2}}+\frac{y^{2}}{b^{2}}=1. \end{aligned}$$

The following expression gives the stress-strain relationship of the Carreau fluid model^16,17:^

9 $$\begin{aligned} \Lambda _{ij}=\mu \left( 1+\left( \Gamma \dot{\gamma }\right) ^{2}\right) ^{\frac{m-1}{2}}\dot{\gamma }_{ij}, \end{aligned}$$

where *m* represents the flow behaviour index, $\Gamma$ is time constant, and

$$\begin{aligned} \dot{\gamma }=\sqrt{\frac{1}{2}\sum _{i}^{}\sum _{j}^{}\dot{\gamma }_{ij}\dot{\gamma }_{ij}}. \end{aligned}$$

On assuming $\Gamma \dot{\gamma }<<1$, we can have

10 $$\begin{aligned} \Lambda _{ij}=\mu \left[ 1+\left( \frac{m-1}{2}\right) \left( \Gamma \dot{\gamma }\right) ^{2}\right] \dot{\gamma }_{ij}. \end{aligned}$$

In the laboratory frame, the fluid flow is unsteady, but it is considered a steady flow in the wave frame (moving frame). The following transformations relate the laboratory and wave frames:

11 $$\begin{aligned} X=x,\,\,\,\,\,\,\,\,Y=y,\,\,\,\,\,\,\,\,Z=z+ct,\,\,\,\,\,\,\,\,U=u,\,\,\,\,\,\,\,\,V=v,\,\,\,\,\,\,\,\,W=w+c. \end{aligned}$$

To transform the equations into non-dimensional form, the adequate dimensionless variables are considered as follows:

12 $$\begin{aligned} \bar{x}&=\frac{x}{D_h},\,\,\,\bar{y}=\frac{y}{D_h},\,\,\,\bar{z}=\frac{z}{\lambda },\,\,\, \bar{u}=\frac{\lambda u}{ c D_h},\,\,\,\bar{v}=\frac{\lambda v}{ c D_h},\,\,\,\delta =\frac{b_0}{a_0}, \,\,\,W_e=\frac{\Gamma u_0}{D_h}, \nonumber \\ \bar{w}&=\frac{w}{c },\,\,\,\bar{p}=\dfrac{D_h^{2}p}{\mu \lambda c },\,\,\,\bar{a}=\bar{a}(z)=\frac{a}{D_h},\,\,\,\bar{b}=\bar{b}(z) =\frac{b}{D_h},\,\,\,\phi =\frac{d}{b_0},\nonumber \\ \bar{t}&=\frac{c t}{\lambda },\,\,\, \bar{\Lambda }_{ij}=\dfrac{D_h }{\mu c} \Lambda _{ij}\,\,\,,\bar{\dot{\gamma }}=\frac{D_h \,\, \dot{\gamma }}{u_0}, \,\,\,\theta =\frac{T-T_w}{T_b-T_w},\,\,\,B_r=\frac{\mu u_{0}^{2}}{k\left( T_b-T_w\right) }, \end{aligned}$$

where $D_h=\frac{b_0\pi }{E(e)}$ is hydraulic diameter of ellipse; $E(e)=\int _{0}^{\frac{\pi }{2}}\sqrt{1-e^2\sin \left( \alpha \right) }\,\,d\alpha$ and $e=\sqrt{1-\delta ^2}$ represents the eccentricity of ellipse such that $0<e<1$.

By employing dimensionless variables provided in (12) together with the long wavelength approximation on Eqs. (1)–(8) and neglecting the dash notation, we obtain

13 $$\begin{aligned} \partial _x p&=0,\,\,\,\,\,\,\,\,\,\,\, (p \,\, \text {is independent of }x) \end{aligned}$$

14 $$\begin{aligned} \partial _y p&=0,\,\,\,\,\,\,\,\,\,\,\, (p \,\,\text {is independent of }y) \end{aligned}$$

15 $$\begin{aligned} p'(z)&=\partial _{x}\Lambda _{xz}+\partial _{y}\Lambda _{yz} , \end{aligned}$$

16 $$\begin{aligned} \left( \partial _{xx}+\partial _{yy}\right) \theta&=-B_r\left( \Lambda _{xz} \partial _x+\Lambda _{yz} \partial _y\right) w . \end{aligned}$$

The dimensionless form of boundary conditions becomes

17 $$\begin{aligned} w=-1\,\,\,\,\,\,\, \text {at}\,\,\,\,\,\, \frac{x^2}{a^2}+\frac{y^2}{b^2}=1, \end{aligned}$$

18 $$\begin{aligned} \theta =0\,\,\,\,\,\,\,\,\,\,\,\,\,\,\text {at}\,\,\,\,\,\, \frac{x^2}{a^2}+\frac{y^2}{b^2}=1. \end{aligned}$$

The non-dimensional mathematical representation of a sinusoidally deformed wall becomes

19 $$\begin{aligned} b&=\frac{E(e)}{\pi } \left( 1+\phi \sin \left( 2\pi z\right) \right) , \end{aligned}$$

20 $$\begin{aligned} a&=3\,\, b. \end{aligned}$$

We acquire the stress components $\Lambda _{xz}$ and $\Lambda _{yz}$ from Eq. (10) as

21 $$\begin{aligned} \Lambda _{xz}&=\mathcal {C}\left( \partial _x w\right) , \end{aligned}$$

22 $$\begin{aligned} \Lambda _{yz}&=\mathcal {C}\left( \partial _y w\right) , \end{aligned}$$

where

$$\begin{aligned} \mathcal {C}=\left[ 1+W_e^{2}\left( \frac{m-1}{2}\right) \left( \left( \partial _x w\right) ^{2}+\left( \partial _y w\right) ^{2}\right) \right] . \end{aligned}$$

By substituting Eqs. (21) and (22) in Eqs. (15) and (16), we acquire

23 $$\begin{aligned} p'(z)&=\partial _{xx}w+\partial _{yy}w+W_e^{2}\left( \frac{m-1}{2}\right) \left[ \partial _x\left( \partial _{x}w\right) ^3+\partial _y\left( \partial _{y}w\right) ^3\right. \nonumber \\&\left. +\partial _x\left( \partial _x w\left( \partial _y w\right) ^2\right) +\partial _y\left( \partial _y w\left( \partial _x w\right) ^2\right) \right] , \end{aligned}$$

24 $$\begin{aligned} \partial _{xx}\,\theta +\partial _{yy}\,\theta =-B_r\left( \mathcal {C}\left( \partial _x w\right) ^2 +\mathcal {C}\left( \partial _y w\right) ^2\right) . \end{aligned}$$

## Solution method

This section consists of the solutions of axial velocity *w*(*x*, *y*) and temperature $\theta (x,y)$.

### Axial velocity

We solve Eq. (23) and (17) by applying perturbation technique via polynomial and considering $W_e^{2}$ as perturbation parameter. Consider

25 $$\begin{aligned} w=w_0+W_e^{2} w_1+\cdot \cdot \cdot , \end{aligned}$$

26 $$\begin{aligned} p=p_0+W_e^{2} p_1+\cdot \cdot \cdot ,\end{aligned}$$

27 $$\begin{aligned} Q=Q_0+W_e^{2} Q_1+\cdot \cdot \cdot . \end{aligned}$$

By using Eqs. (25)-(27) into Eqs. (17) and (23), then equating the coefficients of $\left( W_e^{2}\right) ^0$, $\left( W_e^{2}\right) ^1$, we get the following system:

28 $$\begin{aligned}&\left( W_e^{2}\right) ^0:\,\,\,\,\,\,\,\,\,\,\,\,\,\, {\left\{ \begin{array}{ll} p_0^{'}=\partial _{xx}w_0+\partial _{yy}w_0,\\ \\ \,\,\,\, w_0=-1\,\,\,\,\,\,\ \text {at}\,\,\,\,\,\, \frac{x^2}{a^2}+\frac{y^2}{b^2}=1, \end{array}\right. } \end{aligned}$$

29 $$\begin{aligned}&\left( W_e^{2}\right) ^1:\,\,\,\,\,\,\,\,\,\,\,\,\,\,\,\,\,\,\, {\left\{ \begin{array}{ll} p_1^{'}=\partial _{xx}w_1+\partial _{yy}w_1+\left( \frac{m-1}{2}\right) \left[ \partial _x\left( \partial _{x}w_0\right) ^3+\partial _y\left( \partial _{y}w_0\right) ^3\right. \\ \left. +\partial _x\left( \partial _x w_0\left( \partial _y w_0\right) ^2\right) +\partial _y\left( \partial _y w_0\left( \partial _x w_0\right) ^2\right) \right] ,\\ \\ \,\,\,\, w_1=0\,\,\,\,\,\,\ \text {at}\,\,\,\,\,\, \frac{x^2}{a^2}+\frac{y^2}{b^2}=1, \end{array}\right. } \end{aligned}$$

and neglecting the higher powers of $W_e^{2}$.

Let the following fourth-degree polynomial be the solution of (28)

30 $$\begin{aligned} w_0(x,y)=F_1 x^4+ F_2 y^4+ F_3 x^2 + F_4 y^2 + F_5 x^2 y^2 +F_6. \end{aligned}$$

Then, by using Eq. (30) in Eq. (28) and equating the coefficients of like powers, we have

31 $$\begin{aligned} 6F_1+F_5=0, \end{aligned}$$

32 $$\begin{aligned} 6F_2+F_5=0,\end{aligned}$$

33 $$\begin{aligned} p'_0(z)=2F_3+2F_4,\end{aligned}$$

34 $$\begin{aligned} F_1+\frac{b^4 F_2}{a^4}-\frac{b^2 F_5}{a^2}&=0,\end{aligned}$$

35 $$\begin{aligned} -\frac{2 b^4 F_2}{a^2}+F_3-\frac{b^2 F_4}{a^2}+b^2 F_5&=0 ,\end{aligned}$$

36 $$\begin{aligned} b^4 F_2 + b^2 F_4+ F_6&=-1. \end{aligned}$$

By solving Eqs. (31)–(36) simultaneously, we obtain the values of constants involved in Eq. (30) given in “Appendix”. Therefore, Eq. (30), becomes

37 $$\begin{aligned} w_0(x,y)=\frac{a^2 b^2 p_0^{'}(z)}{2(a^2+b^2)}\left( \frac{x^2}{a^2}+\frac{y^2}{b^2}-1\right) -1, \end{aligned}$$

which is solution of (28). By integrating Eq. (37) over the elliptical cross-section, we get the expression for volumetric flow rate

$$\begin{aligned} q_0(z)=-\frac{a^3 b^3\pi \,\,p_0^{'}(z)}{4(a^2+b^2)}-\pi a b . \end{aligned}$$

The mathematical expression for the pressure gradient $p'_0(z)$ is obtained as

38 $$\begin{aligned} p_0^{'}(z)=-\frac{4 (a^2+b^2)\left( Q_0-L+\pi ab\right) }{a^3 b^3 \pi }, \end{aligned}$$

where $$L=\int _0^1a\, b\, dz.$$

By using a similar procedure we used to solve (28), we obtained the solution of (29) as:

39 $$\begin{aligned} w_1(x,y)&=-\frac{a^2b^2}{12 (a^2+b^2)^4 (a^4+6a^2b^2+b^4)}\left( \frac{x^2}{a^2}+\frac{y^2}{b^2}-1\right) \nonumber \\&\quad \times \left[ 3b^{10}\left( -4p'_1(z)+\left( m-1\right) \left( p'_0(z)\right) ^3x^2\right) +a^2b^8 \left( -108p'_1(z)\left( m-1\right) \left( p'_0(z)\right) ^3\right. \right. \nonumber \\&\quad \times \left. \left. \left( 3b^2+22x^2-3y^2\right) \right) +3a^{10}\left( -4p'_1(z) +\left( m-1\right) \left( p'_0(z)\right) ^3\left( b^2+y^2\right) \right) \right. \nonumber \\&\quad +\,\left. 2a^4b^6\left( -132p'_1(z)+\left( m-1\right) \left( p'_0(z)\right) ^3\left( 8b^2+12x^2+y^2\right) \right) \right. \nonumber \\&\quad +\,\left. 2a^6b^4\left( -132p'_1(z)+\left( m-1\right) \left( p'_0(z)\right) ^3\left( 5b^2+x^2+12y^2\right) \right) \right. \nonumber \\&\quad +\,\left. a^8b^2\left( -108p'_1(z)\left( m-1\right) \left( p'_0(z)\right) ^3 \left( 16b^2-3x^2+22y^2\right) \right) \right] , \end{aligned}$$

40 $$\begin{aligned}&q_1(z)=-\frac{\pi a^3b^3\left[ a^2b^2\left( 3a^4+2a^2b^2+3b^4\right) \left( m-1\right) \left( p'_0(z)\right) ^3-6\left( a^2+b^2 \right) ^3p'_1(z)\right] }{24\left( a^2+b^2\right) ^4}, \end{aligned}$$

41 $$p_{1}^{\prime } (z) = - \frac{{48\left( {a^{2} + b^{2} } \right)^{4} \left( {Q_{1} - L} \right) - \pi a^{5} b^{5} \left( {m - 1} \right)\left( {3a^{4} + 2a^{2} b^{2} + 3b^{4} } \right)\left( {p_{0}^{\prime } (z)} \right)^{3} }}{{12\pi a^{3} b^{3} \left( {a^{2} + b^{2} } \right)^{3} }}.$$

Therefore, we finally acquire the solution of velocity as:

42 $$\begin{aligned} w(x,y)&=-1+\frac{a^2 b^2p'(z)}{2(a^2+b^2)}\left( \frac{x^2}{a^2}+\frac{y^2}{b^2}-1\right) +\frac{1}{3 a^9 b^9 \left( a^2+b^2\right) \left( a^4+6 a^2 b^2+b^4\right) \pi ^3}\nonumber \\&\quad \times \left[ 8 W_e^2(m-1) (\pi a b +Q-L)^3\left( -22 a^2 b^{10} x^4-3 b^{12} x^4+3 a^{12}\left( b^4-y^4\right) \right. \right. \nonumber \\&\quad \left. \left. +\,3 a^4 b^8 \left( b^4-8 x^4-8 x^2 y^2+y^4+2 b^2 \left( x^2-y^2\right) \right) +2 a^6 b^6 \left( 8 b^4-x^4\right. \right. \right. \nonumber \\&\quad \left. \left. \left. -\,24 x^2 y^2-y^4+7 b^2 \left( x^2-y^2\right) \right) +2 a^{10} \left( 8 b^6-11 b^2 y^4-3 b^4 \left( x^2-y^2\right) \right) \right. \right. \nonumber \\&\quad \left. \left. +\,a^8 \left( 10 b^8-14 b^6 \left( x^2-y^2\right) +3 b^4 \left( x^4-8 x^2 y^2-8 y^4\right) \right) \right) \right] . \end{aligned}$$

The following equation is used to find the mathematical result for pressure-gradient:

43 $$\begin{aligned} p'(z)&=\frac{4}{3 a^7 b^7 \pi ^3}\left[ -12 \pi a^3 b^3 (m-1) \left( b^4 \pi ^2+2 (L-Q)^2\right) W_e^2+4 a^2 b^2 (-1+m)\right. \nonumber \\&\quad \left. \times \left( 9 b^4 \pi ^2+2 (L-Q)^2\right) (L-Q) W_e^2-36 a b^5 (-1+m) \pi (L-Q)^2 W_e^2\right. \nonumber \\&\quad \left. +\,12 b^4 (m-1) (L-Q)^3 W_e^2-3 a^7 b^3 \pi ^3 \left( b^2+4 (-1+m) W_e^2\right) -a^5 b \pi \left( 3 b^6 \pi ^2\right. \right. \nonumber \\&\quad \left. \left. +\,4 (m-1) \left( 2 b^4 \pi ^2+9 (L-Q)^2\right) W_e^2\right) +3 a^6 b^2 \pi ^2 \left( 12 (m-1) (L-Q) W_e^2\right. \right. \nonumber \\&\quad \left. \left. +\,b^2 \left( L-Q+L W_e^2\right) \right) +3 a^4 \left( 4 (m-1) \left( 2 b^4 \pi ^2+(L-Q)^2\right) (L-Q) W_e^2\right. \right. \nonumber \\&\quad \left. \left. +\,b^6 \pi ^2 \left( L-Q+L W_e^2\right) \right) \right] . \end{aligned}$$

The mathematical expression of pressure-rise for one wavelength can be attained by

44 $$\begin{aligned} \Delta p=\int _{0}^{1}p'(z) dz. \end{aligned}$$

### Temperature distribution

For the solution of temperature, we consider the following polynomial as the solution of Eq. (24):

45 $$\begin{aligned} \theta (x,y)&=x^2 c_1+x^4 c_2+x^6 c_3+y^2 c_4+y^4 c_5+y^6 c_6+c_7+\left( x^2 y^2 \right) c_8+x^4 y^4 c_9\nonumber \\&\quad +\left( x^4 y^2-x^2 y^4\right) c_{10}+c_{11}\left( x^4 y^2+x^2 y^4\right) +c_{13}\left( x^6 y^4-x^4 y^6\right) +c_{14}x^8 \nonumber \\&\quad +\,c_{15}y^8+c_{16}x^6y^6+c_{17}x^8y^8+\left( x^6 y^2-x^2 y^6\right) c_{18}+c_{19}\left( x^6 y^2+x^2 y^6\right) \nonumber \\&\quad +\left( x^8 y^2-x^2 y^8\right) c_{20}+c_{21}\left( x^8 y^2+x^2 y^8\right) +\left( x^8 y^4-x^4 y^8\right) c_{22}+c_{23}\left( x^8 y^4\right. \nonumber \\&\quad \left. +\,x^4 y^8\right) +c_{24}\left( x^8 y^6-x^6 y^8\right) +c_{25}x^{10}+c_{26}y^{10}+c_{27}x^{12}+c_{28}y^{12}+c_{29}x^{10}y^{10}\nonumber \\&\quad +\,c_{30}\left( x^{10} y^2-x^2 y^{10}\right) +c_{31}\left( x^{10} y^2+x^2 y^{10}\right) +c_{32}\left( x^{10} y^4-x^4 y^{10}\right) +c_{33}\left( x^{10} y^4\right. \nonumber \\&\quad \left. +\,x^4 y^{10}\right) +c_{34}\left( x^{10} y^6-x^6 y^{10}\right) +c_{35}\left( x^{10} y^6+x^6 y^{10}\right) +c_{36}\left( x^{10} y^8-x^8 y^{10}\right) \nonumber \\&\quad +\,c_{37}\left( x^{10} y^8+x^8 y^{10}\right) +c_{38}\left( x^8 y^6+x^6 y^8\right) . \end{aligned}$$

By utilizing the similar process as we adopted for the solution of $w_0(x,y)$, we evaluate the constants $c_l$’s where $l=1,2,...,38$ and attain the solution of temperature. The values of constants $c_l$’s are provided in the “Appendix”.

## Special cases and validation

- By setting $a=b$, we attain the axial velocity for Carreau fluid flow through a circular cross-section duct.
- On substituting $m=1$ or $\Gamma =0$, we get the mathematical expression for the Newtonian fluid flow through a duct of elliptical cross-section given by McCash et al. (with $\phi _1=\phi _2=0$) in^18^.

The solutions of axial velocity and temperature satisfy the PDEs and the boundary conditions. The graphs of axial velocity and temperature also satisfy the boundary conditions. It assures and guarantees the validation of our results. Further, Table 1 also ensures the validation of the results presented in this work.

**Table 1:** Comparison table of numerical values for velocity and temperature with fix parameters $\delta =0.13,\,\phi =0.4,\,z=1,\,Q=0.25,\,B_r=0.35,\,W_e=0,\,m=1$.

*x*	*y*	*w* in present study	$\theta$ in present study	*w* in^18^ with $\phi _1=\phi _2=0$	$\theta$ in^18^ with $\phi _1=\phi _2=0$
0	0	0.90079	0.36574	0.90079	0.36574
0.2	0.05	0.83570	0.35644	0.83570	0.35644
0.4	0.1	0.640431	0.32721	0.640431	0.32721
0.6	0.15	0.31497	0.27389	0.31497	0.27389
0.7	0.2	0.01555	0.22761	0.01555	0.22761

## Results and discussion

The present work aims to analyze the peristaltic flow of Carreau fluid through a duct with an elliptic cross-section. The mathematical equations are solved using the perturbation technique ($W_e^2$ as perturbation parameter) and polynomial solution. This section consists of the graphical investigation of the solutions of mathematical equations obtained in the above fragment. In this section, we examined the effects of different physical parameters on flow characteristics such as flow velocity, pressure gradient, pressure- rise, and temperature distribution. The graphs are plotted by writing the computer programs on Mathematica 13.2.

**Figure 2:**
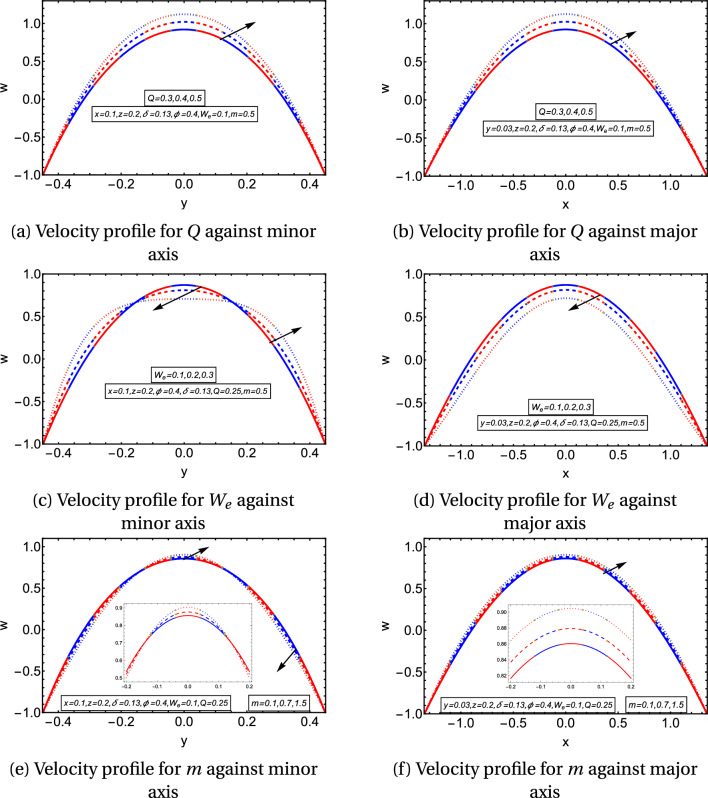
Velocity graphs.

Figures 2a,b give the influence of flow rate on the velocity of flow along the minor and major axes, respectively. The axial velocity *w*(*x*, *y*) gets bigger values for rising flow rate *Q* along both axes. Figures 2c,d indicate the behavior of the Weisenberg number on the flow velocity. It is observed that flow velocity decreases along the major axis with an enhancement in the Weissenberg number. Along the minor axis, it diminishes almost up to $45\%$ length from the center-line, but after this length, the velocity profile reverses and increases in this region. It emphasized that the non-Newtonian effects predominate along the minor axis. Figures 2e,f display the impact of the flow behavior index *m* on the velocity profile. The rising values of *m* cause the enhancement of the flow velocity against the major axis. The graph of flow velocity depicts the similar dual behavior along the minor axis as it did for $W_e$. Moreover, it is noted that the velocity profile is parabolic and axisymmetric. It attains its maximum value at the mid-line of the channel and diminishes towards the boundary to achieve its minimum value at the elliptic boundary.

**Figure 3:**
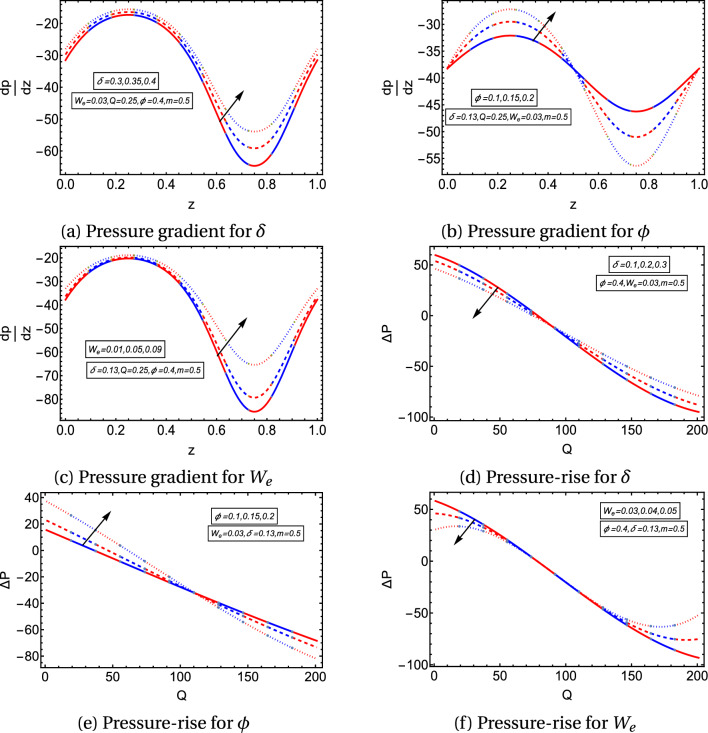
Graphs of pressure gradient and pressure-rise.

Figures 3a–c provide the effects of aspect ratio $\delta$, occlusion $\phi$ and Weissenberg number $W_e$ on the graph of $\frac{dp}{dz}$ against the axial direction (*z*-axis). Figure 3a illustrates that the pressure gradient increases as the aspect ratio $\delta$ increases. The increase in aspect ratio means that the cross-section of the channel becomes less elliptical. Since the decrease in the pressure gradient assists the fluid in flowing along the axial direction. Therefore, it pointed out that the peristaltic flow velocity of the fluid is higher in the highly elliptical channels. Figure 3b delineates that pressure gradient rises and reduces in the expanding and contracting regions of the peristaltic wave, respectively, for higher values of $\phi$. $\frac{dp}{dz}$ gets larger values for growing values of Weissenberg number $W_e$ as shown in Fig. 3c. In Figs. 3d–f, pressure-rise graphs are plotted and examined for the effects of parameters on the positive value region (peristaltic pumping, $\Delta p>0$ ), zero value region (free pumping, $\Delta p=0$ ) and the negative value region (augmented pumping, $\Delta p<0$). Figures 3d,f demonstrate that the pressure-rise lessens in the peristaltic pumping zone. In contrast, it exhibits the opposite behavior in the augmented pumping zone for a higher aspect ratio and Weissenberg number. Figure 3e explains the impact of occlusion $\phi$ on the pressure-rise graph. It depicts that pressure-rise grows for a growing $\phi$ in the peristaltic pumping and free pumping regions, but its behavior reverses in the augmented pumping region.

**Figure 4:**
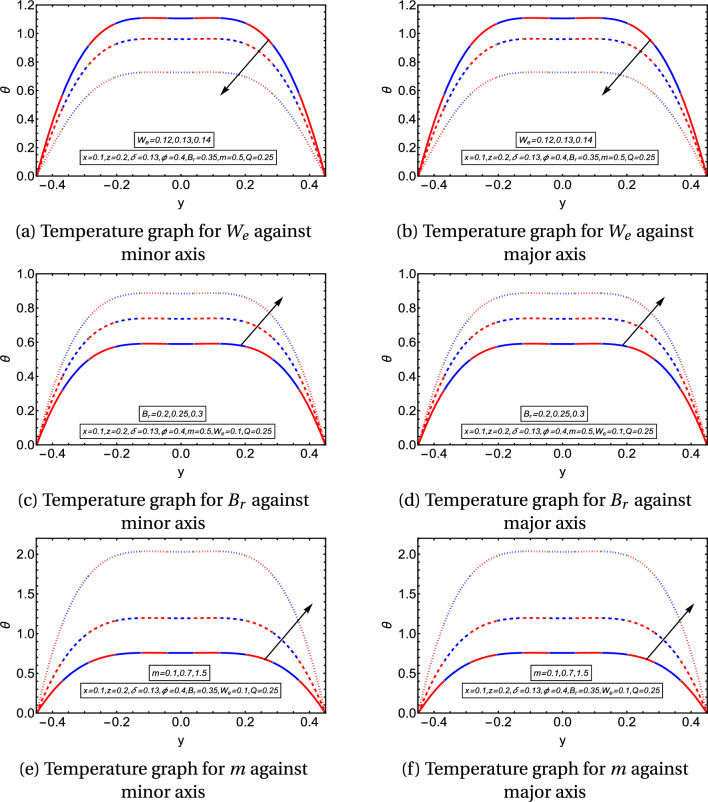
Temperature graphs.

**Figure 5:**
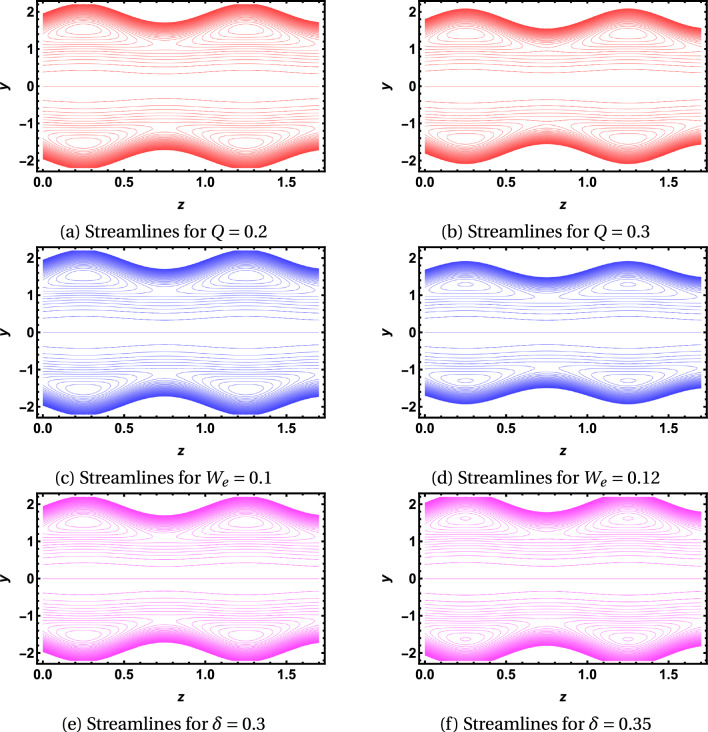
Streamlines.

Figures 4a–f provide the graphical temperature behavior for the flow behavior index, Weissenberg, and Brinkman numbers. Figures 4a,b depict the temperature profile for $W_e$. They delineate that the temperature graph decreases along the minor and major axes. Figures 4c,d describe that the temperature rises with the rising values of $B_r$ along the minor and major axes. The temperature profile enhances by incrementing the values of *m* as provided in Figs. 4e,f. It is important to note from the temperature graphs that they depict a parabolic nature along the major axis, but the parabolic profile is disturbed along the minor axis. It is observed that temperature has the same values in the region $-0.2\le y\le 0.2$ and diminishes quickly in the surrounding wall of the channel along the minor axis, in Figs. 5a–f, streamlines are also plotted to examine the flow behavior. They explain that the streamlines break into contours near the peristaltic moving wall. The contours reduce in numbers for larger *Q* and diminish in both size and count for higher values of $W_e$. In contrast, the contours rise in the count for a higher aspect ratio value.

## Concluding remarks

In this study, we analyzed the flow of Carreau fluid through a duct with an elliptical cross-section. The problem is formulated mathematically in Cartesian coordinates. The resulting differential equations are solved in the dimensionless form under the long wavelength assumption. The momentum equation is solved by employing the perturbation technique via a polynomial solution. The energy equation is solved using a $20^{th}$ degree polynomial. The solutions of mathematical equations are examined graphically. The key findings of our work are provided as follows:

- The flow velocity showed dual behavior along the minor axis of the elliptic duct. It allows us to conclude that non-Newtonian effects become dominant in the conduit of a narrower cross-section.
- The flow velocity is symmetric about the axis of the channel and parabolic in nature. Further, it gains maximum value at the midline.
- It is noted that fluid velocity decreases with the rise in aspect ratio. Therefore, we can conclude that flow velocity is higher in the channels with a high elliptical cross-section.
- The temperature graph along the major axis is perfectly parabolic, but the parabolic nature disturbs the minor axis and reduces quickly in the area surrounding the channel’s boundary. It also assures the dominance of non-Newtonian impact in the narrower cross-section.
- Towards the peristaltic moving wall, it is seen that streamlines transform into contours. It is also noted that the flow is smooth in the mid-region of the elliptic duct.

## Data Availability

The data used to support the findings of this study is included within the article.

## Appendix

$$\begin{aligned} c_1&=-\frac{1}{90 a^3 b^3 \pi ^3 E_1^4 E_2^2 E_3}(a-b) (a+b) B_r \left( p'(z)\right) \left( -1152 b^{16} M^3 W_e^2+3456 a b^{17} M^2 \pi W_e^2\right. \nonumber \\&\left. \quad -\,3456 a^{18} b^2 M \pi ^2 W_e^2+3 a^{19} b^3 \pi ^3 \left( 5 b^4 \left( p'(z)\right) +\left( 384+5 b^4 \left( p'(z)\right) ^3\right) W_e^2\right) +128 a^9 b \pi \right. \nonumber \\&\left. \quad \times\, \left( -\left( 9 a^{14}+120 a^{12} b^2+292 a^{10} b^4-440 a^8 b^6-1242 a^6 b^8-440 a^4 b^{10}+292 a^2 b^{12}\right. \right. \right. \nonumber \\&\left. \left. \left. \quad +\,120 b^{14}\right) M^3+12 a b^{13} \left( 73 a^2+30 b^2\right) M^2 \pi -3 b^4 \left( 120 a^{14}+292 a^{12} b^2-440 a^{10} b^4\right. \right. \right. \nonumber \\&\left. \left. \left. \quad -\,1242 a^8 b^6-440 a^6 b^8+292 a^4 b^{10}+120 a^2 b^{12}+9 b^{14}\right) M \pi ^2+3 a b^{17} \left( 40 a^2+3 b^2\right) \pi ^3\right) \right. \nonumber \\&\left. \quad \times\, W_e^2 \left( 384 \left( 9 a^{10}+120 a^8 b^2+292 a^6 b^4-440 a^4 b^6-1242 a^2 b^8-440 b^{10}\right) M^2 W_e^2\right. \right. \nonumber \\&\left. \left. \quad +\,b^4 \pi ^2 \left( 15 b^4 \left( 22 a^{10}+127 a^8 b^2+212 a^6 b^4+127 a^4 b^6+22 a^2 b^8+b^{10}\right) \left( p'(z)\right) \right. \right. \right. \nonumber \\&\left. \left. \left. \quad +\,256 \left( 60 a^{10}+146 a^8 b^2-220 a^6 b^4-621 a^4 b^6-220 a^2 b^8+146 b^{10}\right) W_e^2\right. \right. \right. \nonumber \\&\left. \left. \left. \quad +\,15 b^4 \left( 20 a^{10}+87 a^8 b^2+40 a^6 b^4+87 a^4 b^6+20 a^2 b^8+b^{10}\right) \left( p'(z)\right) ^3 W_e^2\right) \right) \right) ,\\ c_2&=\left( B_r \left( p'(z)\right) \left( -1920 b^{18} M^3 W_e^2+5760 a b^{19} M^2 \pi W_e^2-6912 a^{20} b^2 M \pi ^2 W_e^2-1152 \right. \right. \nonumber \\&\left. \left. \quad \times\, a^{18} M\left( 2 M^2+5 b^4 \pi ^2\right) W_e^2+384 a^3 b^{17} \pi \left( 398 M^2+5 b^4 \pi ^2\right) W_e^2- 128 a^2 b^{16} M \left( 398 M^2\right. \right. \right. \nonumber \\&\left. \left. \left. \quad +\,45 b^4 \pi ^2\right) W_e^2-256 a^4 b^{14} M \left( 1510 M^2+597 b^4 \pi ^2\right) W_e^2+384 a^{16} b^2 M \left( -5 M^2\right. \right. \right. \nonumber \\&\left. \left. \left. \quad +\,1676 b^4 \pi ^2\right) W_e^2- 512 a^6 b^{12} M \left( 1951 M^2+2265 b^4 \pi ^2\right) W_e^2-768 a^8 b^{10} M \left( 695 M^2\right. \right. \right. \nonumber \\&\left. \left. \left. \quad +\,3902 b^4 \pi ^2\right) W_e^2+512 a^{12} b^6 M \left( 1805 M^2+4908 b^4 \pi ^2\right) W_e^2+ 512 a^{14} b^4 M \left( 419 M^2\right. \right. \right. \nonumber \\&\left. \left. \left. \quad +\,5415 b^4 \pi ^2\right) W_e^2-256 a^{10} b^8 M \left( -3272 M^2+6255 b^4 \pi ^2\right) W_e^2+3 a^{21} b^3 \pi ^3 \left( 5 b^4 \left( p'(z)\right) \right. \right. \right. \nonumber \\&\left. \left. \left. \quad +\,\left( 768+5 b^4 \left( p'(z)\right) ^3\right) W_e^2\right) + 2 a^9 b^{11} \pi \left( -1935 b^8 \left( p'(z)\right) \pi ^2+\left( 800640 M^2+b^4 \left( 499456\right. \right. \right. \right. \right. \nonumber \\&\left. \left. \left. \left. \left. \quad -\,1545 b^4 \left( p'(z)\right) ^3\right) \pi ^2\right) W_e^2\right) + 2 a^7 b^{13} \pi \left( -210 b^8 \left( p'(z)\right) \pi ^2+\left( 1498368 M^2+5 b^4 \left( 38656\right. \right. \right. \right. \right. \nonumber \\&\left. \left. \left. \left. \left. \quad -\,39 b^4 \left( p'(z)\right) ^3\right) \pi ^2\right) W_e^2\right) +a^5 b^{15} \pi \left( -15 b^8 \left( p'(z)\right) \pi ^2+\left( 1159680 M^2+b^4 \left( 50944\right. \right. \right. \right. \right. \nonumber \\&\left. \left. \left. \left. \left. \quad -\,15 b^4 \left( p'(z)\right) ^3\right) \pi ^2\right) W_e^2\right) + 6 a^{19} b \pi \left( 40 b^8 \left( p'(z)\right) \pi ^2+\left( 1152 M^2+5 b^4 \left( 64+7 b^4\right. \right. \right. \right. \right. \nonumber \\&\left. \left. \left. \left. \left.\quad \times\, \left( p'(z)\right) ^3\right) \pi ^2\right) W_e^2\right) -8 a^{13} b^7 \pi \left( 2385 b^8 \left( p'(z)\right) \pi ^2+2 \left( 173280 M^2+b^4 \left( 52352+225 b^4 \right. \right. \right. \right. \right. \nonumber \\&\left. \left. \left. \left. \left. \quad \times\, \left( p'(z)\right) ^3\right) \pi ^2\right) W_e^2\right) - 2 a^{17} b^3 \pi \left( 45 b^8 \left( p'(z)\right) \pi ^2+\left( -2880 M^2+b^4 \left( 107264+255 b^4 \right. \right. \right. \right. \right. \nonumber \\&\left. \left. \left. \left. \left. \quad \times\, \left( p'(z)\right) ^3\right) \pi ^2\right) W_e^2\right) -6 a^{11} b^9 \pi \left( 2380 b^8 \left( p'(z)\right) \pi ^2+\left( 418816 M^2+5 b^4\right. \right. \right. \right. \nonumber \\&\left. \left. \left. \left. \quad \times\, \left( -17792+271 b^4 \left( p'(z)\right) ^3\right) \pi ^2\right) W_e^2\right) - 2 a^{15} b^5 \pi \left( 4290 b^8 \left( p'(z)\right) \pi ^2+\left( 321792 M^2\right. \right. \right. \right. \nonumber \\&\left. \left. \left. \left. \quad +\,5 b^4\left( 92416+753 b^4 \left( p'(z)\right) ^3\right) \pi ^2\right) W_e^2\right) \right) \right) \times\, \frac{1}{180 a^5 b^3 \pi ^3 E_1^4 E_2^2 E_3},\\ c_3&=\frac{64 \left( p'(z)\right) (M-a b \pi )^3 B_r W_e^2}{45 a^9 b^3 \pi ^3 E_1^2 E_2 E_3 }\times\, \left( 3 a^{12}-56 a^{10} b^2-27 a^8 b^4+320 a^6 b^6\right. \nonumber \\&\left. \quad +\,333 a^4 b^8+64 a^2 b^{10}+3 b^{12}\right) ,\\ c_4&=-\,c_1, \end{aligned}$$

$$\begin{aligned} c_5&=\frac{-1}{180 a^3 b^5 \pi ^3 E_1^4 E_2^2 E_3} \left( \left( p'(z)\right) B_r \left( 2304 b^{18} M^3 W_e^2-6912 a b^{19} M^2 \pi W_e^2+5760 a^{20} b^2 \right. \right. \nonumber \\&\left. \left. \quad \times\, M \pi ^2 W_e^2-1152 a^3 b^{17} \pi \left( 5 M^2+2 b^4 \pi ^2\right) W_e^2-384 a^5 b^{15} \pi \left( -1676 M^2+5 b^4 \pi ^2\right) W_e^2\right. \right. \nonumber \\&\left. \left. \quad +\, 384 a^2 b^{16} M \left( 5 M^2+18 b^4 \pi ^2\right) W_e^2+128 a^4 b^{14} M \left( -1676 M^2+45 b^4 \pi ^2\right) W_e^2+384 a^{18}\right. \right. \nonumber \\&\left. \left. \quad \times\, M \left( 5 M^2+398 b^4 \pi ^2\right) W_e^2- 512 a^6 b^{12} M \left( 1805 M^2+1257 b^4 \pi ^2\right) W_e^2-768 a^{10} b^8 M\right. \right. \nonumber \\&\left. \left. \quad \times\, \left( -695 M^2+3272 b^4 \pi ^2\right) W_e^2+256 a^{16} b^2 M \left( 199 M^2+4530 b^4 \pi ^2\right) W_e^2- 512 a^8 b^{10} M \right. \right. \nonumber \\&\left. \left. \quad \times\, \left( 1636 M^2+5415 b^4 \pi ^2\right) W_e^2+512 a^{14} b^4 M \left( 755 M^2+5853 b^4 \pi ^2\right) W_e^2+256 a^{12} b^6 M \right. \right. \nonumber \\&\left. \left. \quad \times\, \left( 3902 M^2+6255 b^4 \pi ^2\right) W_e^2+ 15 a^{23} b^5 \left( p'(z)\right) \pi ^3 \left( 1+\left( p'(z)\right) ^2 W_e^2\right) +30 a^{21} b^3 \pi ^3 \right. \right. \nonumber \\&\left. \left. \quad \times\, \left( 14 b^4 \left( p'(z)\right) +\left( -64+13 b^4 \left( p'(z)\right) ^3\right) W_e^2\right) +8 a^{15} b^5 \pi \left( 2385 b^8 \left( p'(z)\right) \pi ^2-2 72480\right. \right. \right. \nonumber \\&\left. \left. \left. \quad \times\, M^2W_e^2-2b^4 \left( 62432-225 b^4 \left( p'(z)\right) ^3\right) \pi ^2W_e^2 \right) + 2 a^9 b^{11} \pi \left( -120 b^8 \left( p'(z)\right) \pi ^2\right. \right. \right. \nonumber \\&\left. \left. \left.\quad +\left( 1256448 M^2+5 b^4 \left( 92416-21 b^4 \left( p'(z)\right) ^3\right) \pi ^2\right) W_e^2\right) + a^7 b^{13} \pi \left( -15 b^8 \left( p'(z)\right) \pi ^2\right. \right. \right. \nonumber \\&\left. \left. \left. \quad +\,\left( 2772480 M^2+b^4 \left( 214528-15 b^4 \left( p'(z)\right) ^3\right) \pi ^2\right) W_e^2\right) + 6 a^{13} b^7 \pi \left( 1430 b^8 \left( p'(z)\right) \pi ^2\right. \right. \right. \nonumber \\&\left. \left. \left. \quad +\,\left( -499456 M^2+5 b^4 \left( -17792+251 b^4 \left( p'(z)\right) ^3\right) \pi ^2\right) W_e^2\right) + 2 a^{11} b^9 \pi \left( 45 b^8 \left( p'(z)\right) \pi ^2\right. \right. \right. \nonumber \\&\left. \left. \left. \quad +\,\left( -800640 M^2+b^4 \left( 418816+255 b^4 \left( p'(z)\right) ^3\right) \pi ^2\right) W_e^2\right) + 2 a^{17} b^3 \pi \left( 7140 b^8 \left( p'(z)\right) \pi ^2\right. \right. \right. \nonumber \\&\left. \left. \left. \quad +\,\left( -76416 M^2+5 b^4 \left( -38656+813 b^4 \left( p'(z)\right) ^3\right) \pi ^2\right) W_e^2\right) + 2 a^{19} b \pi \left( 1935 b^8 \left( p'(z)\right) \pi ^2\right. \right. \right. \nonumber \\&\left. \left. \left. \quad +\,\left( -2880 M^2+b^4 \left( -25472+1545 b^4 \left( p'(z)\right) ^3\right) \pi ^2\right) W_e^2\right) \right) \right) ,\\ c_6&=\frac{64 \left( p'(z)\right) (M-a b \pi )^3 B_r W_e^2}{45 a^3 b^9 \pi ^3 E_1^2 E_2 E_3 }\times\, \left( 3 a^{12}+64 a^{10} b^2+333 a^8 b^4+320 a^6 b^6-27 a^4 b^8\right. \nonumber \\&\left. \quad -\,56 a^2 b^{10}+3 b^{12}\right) \\ c_7&=\frac{1}{180 a^3 b^3 E_1^4 E_2^2 E_3 \pi ^3}\left( \left( p'(z)\right) B_r \left( -768 b^{20} M^3 W_e^2+2304 a b^{21} M^2 \pi W_e^2-2304 a^{22} b^2 \right. \right. \nonumber \\&\left. \left. \quad \times\, M \pi ^2 W_e^2+15 a^{23} b^7 \left( p'(z)\right) \pi ^3+ a^2 \left( -128 \left( 6 a^{18}+143 a^{16} b^2+1154 a^{14} b^4+4444 a^{12} b^6\right. \right. \right. \right. \nonumber \\&\left. \left. \left. \left. \quad +\,9080 a^{10} b^8+11306 a^8 b^{10}+9080 a^6 b^{12}+4444 a^4 b^{14}+1154 a^2 b^{16}+143 b^{18}\right) M^3\right. \right. \right. \nonumber \\&\left. \left. \left. \quad +\, 384 a b^{17} \left( 1154 a^2+143 b^2\right) M^2 \pi - 384 b^4 \left( 143 a^{18}+1154 a^{16} b^2+4444 a^{14} b^4\right. \right. \right. \right. \nonumber \\&\left. \left. \left. \left. \quad +\,9080 a^{12} b^6+11306 a^{10} b^8+9080 a^8 b^{10}+4444 a^6 b^{12}+1154 a^4 b^{14}+143 a^2 b^{16}\right. \right. \right. \right. \nonumber \\&\left. \left. \left. \left. \quad +\,6 b^{18}\right) M \pi ^2+ a b^3 \left( 18304 a^2 b^{18}+768 b^{20}+3 a^{20} \left( 256+5 b^4 \left( p'(z)\right) ^3\right) \right) \pi ^3\right) W_e^2+ a^7 b \pi \right. \right. \nonumber \\&\left. \left. \quad \times\, \left( 15 b^8 \left( 26 a^{14}+216 a^{12} b^2+742 a^{10} b^4+1102 a^8 b^6+742 a^6 b^8+216 a^4 b^{10}+26 a^2\right. \right. \right. \right. \nonumber \\&\left. \left. \left. \left. \quad \times\, b^{12}+b^{14}\right) \left( p'(z)\right) \pi ^2+ \left( 384 \left( 6 a^{14}+143 a^{12} b^2+1154 a^{10} b^4+4444 a^8 b^6+9080 a^6 b^8\right. \right. \right. \right. 
\right. \nonumber \\&\left. \left. \left. \left. \left. \quad +\,11306 a^4 b^{10}+9080 a^2 b^{12}+4444 b^{14}\right) M^2+ b^4 \left( 128 \left( 143 a^{14}+1154 a^{12} b^2\right. \right. \right. \right. \right. \right. \nonumber \\&\left. \left. \left. \left. \left. \left. \quad +\,4444 a^{10} b^4+9080 a^8 b^6+11306 a^6 b^8+9080 a^4 b^{10}+4444 a^2 b^{12}+1154 b^{14}\right) \right. \right. \right. \right. \right. \nonumber \\&\left. \left. \left. \left. \left. \quad +\, 15 b^4 \left( 24 a^{14}+168 a^{12} b^2+408 a^{10} b^4+334 a^8 b^6+408 a^6 b^8+168 a^4 b^{10}\right. \right. \right. \right. \right. \right. \nonumber \\&\left. \left. \left. \left. \left. \left. \quad +\,24 a^2 b^{12}+b^{14}\right) \left( p'(z)\right) ^3\right) \pi ^2\right) W_e^2\right) \right) \right) , \end{aligned}$$

$$\begin{aligned} c_8&= \frac{-1}{15 a^3 b^3 E_1^4 E_2^2 E_3 \pi ^3}\left( \left( p'(z)\right) B_r \left( -2112 b^{16} M^3 W_e^2+6336 a b^{17} M^2 \pi W_e^2-6336 a^{18} b^2\right. \right. \nonumber \\&\left. \left.\quad \times\, M \pi ^2 W_e^2+192 a^3 b^{15} \pi \left( 380 M^2+11 b^4 \pi ^2\right) W_e^2+256 a^5 b^{13} \pi \left( 723 M^2+95 b^4 \pi ^2\right) W_e^2-\right. \right. \nonumber \\&\left. \left.\quad 64 a^2 b^{14} M \left( 380 M^2+99 b^4 \pi ^2\right) W_e^2+256 a^{12} b^4 M \left( -241 M^2+285 b^4 \pi ^2\right) W_e^2-256 a^4 b^{12} \right. \right. \nonumber \\&\left. \left. \quad \times\, M \left( 241 M^2+285 b^4 \pi ^2\right) W_e^2- 192 a^{16} M \left( 11 M^2+380 b^4 \pi ^2\right) W_e^2+128 a^8 b^8 M\right. \right. \nonumber \\&\left. \left. \quad \times\, \left( 997 M^2+570 b^4 \pi ^2\right) W_e^2-256 a^6 b^{10} M \left( -95 M^2+723 b^4 \pi ^2\right) W_e^2- 256 a^{14} b^2 M\right. \right. \nonumber \\&\left. \left. \quad \times\, \left( 95 M^2+723 b^4 \pi ^2\right) W_e^2+128 a^{10} b^6 M \left( 190 M^2+2991 b^4 \pi ^2\right) W_e^2+3 a^{19} b^3 \pi ^3\right. \right. \nonumber \\&\left. \left. \quad \times\, \left( 5 b^4 \left( p'(z)\right) +\left( 704+5 b^4 \left( p'(z)\right) ^3\right) W_e^2\right) + 2 a^9 b^9 \pi \left( 165 b^8 \left( p'(z)\right) \pi ^2-2 \left( 95712 M^2\right. \right. \right. \right. \nonumber \\&\left. \left. \left. \quad +\,5 b^4 \left( 1216 \left. -15 b^4 \left( p'(z)\right) ^3\right) \pi ^2\right) W_e^2\right) +4 a^{13} b^5 \pi \left( 795 b^8 \left( p'(z)\right) \pi ^2+2 \left( 23136 M^2\right. \right. \right. \right. \nonumber \\&\left. \left. \left. \left. \quad +\,5 b^4 \left( -608+15 b^4 \left( p'(z)\right) ^3\right) \pi ^2\right) W_e^2\right) + 2 a^{17} b \pi \left( 165 b^8 \left( p'(z)\right) \pi ^2+2 \left( 1584 M^2\right. \right. \right. \right. \nonumber \\&\left. \left. \left. \left. \quad +\,5 b^4 \left( 1216+15 b^4 \left( p'(z)\right) ^3\right) \pi ^2\right) W_e^2\right) +a^7 b^{11} \pi \left( 15 b^8 \left( p'(z)\right) \pi ^2+\left( -72960 M^2\right. \right. \right. \right. \nonumber \\&\left. \left. \left. \left.\quad +\,b^4 \left( 61696+15 b^4 \left( p'(z)\right) ^3\right) \pi ^2\right) W_e^2\right) + a^{11} b^7 \pi \left( 1905 b^8 \left( p'(z)\right) \pi ^2+\left( -72960 M^2\right. \right. \right. \right. \nonumber \\&\left. \left. \left. \left. \quad +\,b^4 \left( -127616+1305 b^4 \left( p'(z)\right) ^3\right) \pi ^2\right) W_e^2\right) + a^{15} b^3 \pi \left( 1905 b^8 \left( p'(z)\right) \pi ^2+\left( 72960 M^2\right. \right. \right. \right. \nonumber \\&\left. \left. \left. \left. \quad +\,b^4 \left( 61696+1305 b^4 \left( p'(z)\right) ^3\right) \pi ^2\right) W_e^2\right) \right) \right) ^3\\ c_9&=0,\\ c_{10}&=-\frac{ 64 (a^2-b^2) \left( a^8+24 a^6 b^2+54 a^4 b^4+24 a^2 b^6+b^8\right) \left( p'(z)\right) (M-a b \pi )^3 B_r W_e^2}{3 a^5 b^5\pi ^3 E_1^2E_2E_3},\\ c_{11}&=\frac{64(a^2+b^2)W_e^2B_r\left( p'(z)\right) \left( M-ab\pi \right) ^3}{3 a^5 b^5\pi ^3 E_2}. \\ c_l&=0 \text{ for} \,\,\,l=12,13,...38.\\&\quad M=\left( L-Q\right) ,\,\,\,\,\,\,\,\,\,\,\,\,E_1=\left( a^2+b^2\right) \\&\quad E_2=\left( a^4+6 a^2 b^2+b^4\right) ,\,\,\,\,\,\,\,\,\,\,\,\,E_3=\left( a^4+14 a^2 b^2+b^4\right) \\&F_1=F_2=F_5=0,\,\,\,\,\,\,\,\,\,\,\,\,\,F_3=\frac{b^2}{2E_1}p_0^{'}(z)\\&F_4=\frac{a^2}{2E_1}p_0^{'}(z),\,\,\,\,\,\,\,\,\,\,\,\,\,F_6=-\frac{2E_1+a^2 b^2 p_0^{'}(z)}{2E_1}. \end{aligned}$$

## List of symbols

$$D_h$$: Hydraulic diameter
$$\mu$$: Dynamic viscosity
$a_0,b_0$: Semi-axes of non-deformed ellipse
$$\Gamma$$: Time constant
$$\lambda$$: Wavelength
$$W_e$$: Weissenberg number
$$T_w$$: Wall temperature
*m*: Flow behaviour index
$$\rho$$: Density
$$B_r$$: Brinkman number
$\Lambda$: Extra Stress tensor
$$T_b$$: Bulk temperature
*c*: Propagation velocity
*d*: Wave amplitude
*e*: Eccentricity
*U*, *V*, *W*: Components of velocity
*X*, *Y*, *Z*: Cartesian coordinates
$$\delta$$: Aspect ratio
$$\phi$$: Occlusion

## References

[1] Nadeem, S., Akhtar, S., Alharbi, F. M., Saleem, S. & Issakhov, A. Analysis of heat and mass transfer on the peristaltic flow in a duct with sinusoidal walls: Exact solutions of coupled PDEs. *Alex. Eng. J.* **61**(5), 4107–4117 (2022). 10.1016/j.aej.2021.08.087

[2] Mekheimer, K. S. Effect of the induced magnetic field on peristaltic flow of a couple stress fluid. *Phys. Lett. A* **372**(23), 4271–4278 (2008). 10.1016/j.physleta.2008.03.059

[3] Nadeem, S. & Akram, S. Peristaltic flow of a Williamson fluid in an asymmetric channel. *Commun. Nonlinear Sci. Numer. Simul.* **15**(7), 1705–1716 (2010). 10.1016/j.cnsns.2009.07.026

[4] Tripathi, D. & Beg, O. A. A study on peristaltic flow of nanofluids: Application in drug delivery systems. *Int. J. Heat Mass Transf.* **70**, 61–70 (2014). 10.1016/j.ijheatmasstransfer.2013.10.044

[5] Ashraf, H., Siddiqui, A. M. & Rana, M. A. Analysis of the peristaltic-ciliary flow of Johnson–Segalman fluid induced by peristalsis-cilia of the human fallopian tube. *Math. Biosci.* **300**, 64–75 (2018). 29571813 10.1016/j.mbs.2018.03.018

[6] Tripathi, D. A mathematical model for the peristaltic flow of chyme movement in small intestine. *Math. Biosci.* **233**(2), 90–97 (2011). 21802431 10.1016/j.mbs.2011.06.007

[7] Ellahi, R., Bhatti, M. M. & Vafai, K. Effects of heat and mass transfer on peristaltic flow in a non-uniform rectangular duct. *Int. J. Heat Mass Transf.* **71**, 706–719 (2014). 10.1016/j.ijheatmasstransfer.2013.12.038

[8] Zeeshan, A., Ijaz, N., Bhatti, M. M. & Mann, A. B. Mathematical study of peristaltic propulsion of solid-liquid multiphase flow with a biorheological fluid as the base fluid in a duct. *Chin. J. Phys.* **55**(4), 1596–1604 (2017). 10.1016/j.cjph.2017.05.020

[9] Nadeem, S., Qadeer, S., Akhtar, S., El Shafey, A. M. & Issakhov, A. Eigenfunction expansion method for peristaltic flow of hybrid nanofluid flow having single-walled carbon nanotube and multi-walled carbon nanotube in a wavy rectangular duct. *Sci. Prog.* **104**(4), 00368504211050292 (2021). 34738839 10.1177/00368504211050292 PMC10358559

[10] McCash, L. B. *et al.* Novel idea about the peristaltic flow of heated Newtonian fluid in elliptic duct having ciliated walls. *Alex. Eng. J.* **61**(4), 2697–2707 (2022). 10.1016/j.aej.2021.07.035

[11] Hussain, Z. *et al.* A mathematical model for radiative peristaltic flow of Jeffrey fluid in curved channel with Joule heating and different walls: Shooting technique analysis. *Ain Shams Eng. J.* **13**(5), 101685 (2022). 10.1016/j.asej.2021.101685

[12] Shahzad, M. H. & Awan, A. U. Mechanics of heated Rabinowitsch fluid in elliptic vertical duct: Peristalsis and analytical study. *Int. J. Mod. Phys. B* **8**, 2350274 (2023). 10.1142/S0217979223502740

[13] Saleem, A. *et al.* Mathematical computations for peristaltic flow of heated non-Newtonian fluid inside a sinusoidal elliptic duct. *Phys. Scr.* **95**(10), 105009 (2020). 10.1088/1402-4896/abbaa3

[14] Rachid, H., Ouazzani, M. T. & Lahlou, N. Entropy generation and mechanical efficiency in laminar peristaltic flow through an elliptical duct. *Heat Transf.* **50**(8), 8525–8539 (2021). 10.1002/htj.22288

[15] Akhtar, S. *et al.* Analytical solutions of PDEs by unique polynomials for peristaltic flow of heated Rabinowitsch fluid through an elliptic duct. *Sci. Rep.* **12**(1), 12943 (2022). 35902642 10.1038/s41598-022-17044-y PMC9334600

[16] Akbar, N. S. & Nadeem, S. Carreau fluid model for blood flow through a tapered artery with a stenosis. *Ain Shams Eng. J.* **5**(4), 1307–1316 (2014). 10.1016/j.asej.2014.05.010

[17] Ahmad, R. *et al.* An analytical approach to study the blood flow over a nonlinear tapering stenosed artery in flow of Carreau fluid model. *Complex.* **2021**, 1–11 (2021).

[18] McCash, L. B., Akhtar, S., Nadeem, S. & Saleem, S. Entropy analysis of the peristaltic flow of hybrid nanofluid inside an elliptic duct with sinusoidally advancing boundaries. *Entropy* **23**(6), 732 (2021). 34207522 10.3390/e23060732 PMC8227057

